# Cracking the Whippet: The Inconsistent Treatment of Myeloneuropathy Secondary to Chronic Nitrous Oxide Misuse

**DOI:** 10.7759/cureus.52978

**Published:** 2024-01-26

**Authors:** Rehman S Crisp

**Affiliations:** 1 Emergency Department, Queen Elizabeth Hospital Birmingham, Birmingham, GBR

**Keywords:** functional vitamin b12 deficiency, neurotoxicity, nitrous oxide abuse, nitrous oxide toxicity, nitrous oxide myelopathy

## Abstract

Background

The recreational abuse of nitrous oxide (N_2_O) is becoming increasingly prevalent within the United Kingdom and across the globe. Chronic abuse can cause nerve and spinal cord damage through the functional inactivation of vitamin B12. We present six cases from a single centre in the United Kingdom between 2016 and 2020 with neurological complications from N_2_O abuse, ranging from paraesthesia to subacute combined degeneration of the spinal cord.

Methodology

A retrospective review of all neurology admissions to Queen Elizabeth Hospital Birmingham (QEHB) between 2016 and 2020 was conducted to identify patients admitted with a diagnosis of neurological dysfunction (neuropathy or myelopathy) in association with chronic N_2_O misuse. The Prescribing Information and Communication System was used to collect data on demographics, reported N_2_O misuse, presenting symptoms, results of blood tests, nerve conduction studies and treatment regimens. All patients gave verbal consent for inclusion in the case series. Data were anonymised and analysed by the corresponding author.

Results

All patients were males aged between 22 and 28 years. In all cases, the patients were admitted with the abuse of N_2_O *whippet* canisters (ranging from 20 to 500 canisters per session), presenting with a combination of sensory and motor disturbance. Clinical suspicion, in the context of a history of N_2_O abuse, along with elevated blood concentrations of methylmalonic acid (MMA) and homocysteine, and nerve conduction studies, was the cornerstone of the diagnosis.

All patients were treated with parenteral vitamin B12, though individual regimens differed, with no standardisation in the duration or frequency of treatment. All patients received intramuscular (IM) vitamin B12 injections during admission, with one patient receiving oral vitamin B12 before being switched to IM vitamin B12 injections. One patient received additional folic acid as a treatment adjunct. Prescriptions were most varied on discharge with huge discrepancies in duration and frequency of vitamin B12 replacement, ranging from no B12 replacement at all to IM injections once weekly for eight weeks.

Conclusions

The variability in route, dose and duration of vitamin B12 treatment, along with the variable use of adjunctive therapy reported in the literature, highlights the current lack of consensus in managing N_2_O neurotoxicity.

## Introduction

First synthesised in 1772, nitrous oxide (N_2_O) was used recreationally at Victorian laughing gas shows due to its euphoric and pleasurable effects, long before its discovery as an anaesthetic. N_2_O is currently the third most popular recreational drug in the United Kingdom, with the prevalence of its use on the rise [1].

The neurological damage associated with N_2_O abuse is largely caused by a functional deficiency of vitamin B12 (Cobalamin), which is inactivated at a cellular level. N_2_O oxidises cobalt(I) in vitamin B12 to cobalt(II), irreversibly inactivating it and resulting in functional vitamin B12 deficiency. Vitamin B12 acts as a co-factor for methionine synthase in its conversion of homocysteine into methionine, which is required for the methylation of myelin protein production. By inhibiting methionine synthase and thus affecting the ability to produce myelin proteins, the functional deficiency of vitamin B12 due to N2O effectively leads to defective myelination of nerves in both the central and peripheral nervous systems [2,3]. Repeated exposure can result in various clinical manifestations, the most common of which are neurological, ranging from paraesthesia to subacute degeneration of the cord (SCDC). Up to 3.3% of chronic users develop neurological damage [4]. 

Despite the increasing prevalence of N_2_O misuse and its neurological sequelae, no consensus exists on the optimal treatment regimen. We present a case series of six patients admitted to QEHB, United Kingdom, to highlight the inconsistencies in treatment.

## Materials and methods

A retrospective review of all neurology admissions to QEHB between 2016 and 2020 was conducted to identify patients admitted with a diagnosis of neurological dysfunction (neuropathy or myelopathy) in association with chronic N_2_O misuse.

The Prescribing Information and Communication System (PICS) was used to collect data on demographics, reported N_2_O misuse, presenting symptoms, results of blood tests, nerve conduction studies, and treatment regimens. For all patients admitted under neurology between 2016 and 2020, a modified electronic note search was conducted to identify individuals admitted with documented N_2_O abuse or admission with neurological symptoms and associated documentation of N_2_O use. This was done through a manual keyword search on PICS for the aforementioned patients to include nitrous oxide, nitrous, NO, N_2_O, whippets, canisters, subacute demyelination of the cord, vitamin B12 deficiency, myeloneuropathy and neuropathy. In cases where these keywords were identified, the emergency department clerking and initial neurology assessments were reviewed to identify if these cases were suitable for inclusion in the case series. Inclusion criteria focused on any patient with a documented history of prolonged N_2_O with associated clinical neurological deficits that were not attributable to another cause. 

Once identified, a full review of the admission notes from these patients was completed. Analysis of their neurology ward round entries, drug charts and biochemical markers was undertaken to determine any clinical developments or medication and treatment alterations. A modified Rankin score was determined for each patient during their admission and after reviewing their most recent neurology clinic follow-up letter. 

All patients gave verbal consent for inclusion in the case series. The data were anonymised and analysed by the corresponding author.

## Results

We identified six patients who met the criteria for inclusion in this case series (Table 1). All patients were males in their twenties, with a mean age of 22.8 years. Reported misuse of N_2_O varied from 50 small canisters (*whippets*) a week up to 500 small canisters a day. All patients exhibited some biochemical evidence of functional B12 deficiency. Three patients had low blood B12 concentration, while B12 in the other three cases (50%) was at the low-normal end of the reference range. Three patients had MMA levels measured, and all were markedly raised. Homocysteine levels were measured in five patients, and it was raised in four of the five (80%) patients. Four of the six patients underwent nerve conduction studies, with abnormalities detected in all cases. Three of the six patients received intramuscular (IM) B12 on alternate days for the first two weeks, but there were significant variations in inpatient and discharge prescriptions. One patient received oral cyanocobalamin before switching to IM hydroxocobalamin. One patient received additional folic acid supplementation. Follow-up information (at least 12 months) was available for four of the six patients. Three patients had ongoing symptoms but no significant disability (modified Rankin Scale 1); one patient had ongoing moderate disability (modified Rankin Scale 3).

**Table 1:** Patients admitted to Queen Elizabeth Hospital Birmingham with neurological symptoms secondary to N2O misuse, 2016-2020. Modified Rankin Scale: 0, no symptoms at all; 1, no significant disability despite symptoms; able to carry out all usual duties and activities; 2, slight disability; unable to carry out all previous activities, but able to look after own affairs without assistance; 3, moderate disability; requiring some help, but able to walk without assistance; 4, moderately severe disability; unable to walk and attend to bodily needs without assistance; 5, severe disability; bedridden, incontinent and requiring constant nursing care and attention; 6, dead. MMA, methylmalonic acid; IM, intramuscular; IV, intravenous; N/A, not applicable; N_2_O, nitrous oxide

Case	Age (years)	Gender	N₂O use	Presenting complaint	Vitamin B12 (187-883 ng/L)	MMA (73-271 nmol/L)	Homocysteine (5-15 μmol/L)	Nerve conduction study findings	Treatment regimen	Modified Rankin Scale on admission	Modified Rankin Scale on follow-up
1	22	Male	50-75 canisters/week for six months	Progressive lower limb sensory and motor disturbance	302	9,170	172	Severe sensory motor axonal neuropathy predominantly affecting lower limbs	Initially 1 mg IM hydroxocobalamin three times a week for two weeks and 5 mg folic acid once daily. On discharge, 1 mg IM hydroxocobalamin every other day for three months, followed by 1 mg IM hydroxocobalamin once every two months.	2	1
2	22	Male	125 canisters/week for one year	Reduced mobility, leg weakness and paraesthesia	190	>3,800	11	Prolonged terminal latency, borderline amplitude, mildly slow conduction	Initially, cyanocobalamin 50 μg once a day for two weeks, before being switched to 1 mg IM hydroxocobalamin every other day for a week. On discharge, 1 mg IM hydroxocobalamin every three months.	3	N/A
3	22	Male	500 canisters/day, duration unknown	10 days worsening upper and lower limb weakness and paraesthesia	171	2,289	66.3	N/A	Initially, 1 mg IM hydroxocobalamin every other day for two weeks. On discharge: nil	3	3
4	22	Male	240 canisters/week for over six months	Upper and lower limb weakness, lower limb paraesthesia and numbness	245	N/A	>200	Acute/subacute peripheral motor neuropathy	Initially, 1 mg IM hydroxocobalamin every other day for one week. On discharge, 1 mg IM hydroxocobalamin once every eight weeks.	2	1
5	27	Male	75 canisters/day, duration unknown	Two weeks upper and lower limb weakness with lower limb numbness and paraesthesia	156	N/A	92	Significant pathology within central somatosensory conduction either within the brain or at the level of the dorsal columns	Initially, 1 mg IM hydroxocobalamin for three days. On discharge, 1 mg IM hydroxocobalamin once daily for one week followed by 1 mg IM hydroxocobalamin once weekly for eight weeks.	2	N/A
6	22	Male	*Many* canisters/week for three months	Two days leg weakness, poor mobility and paraesthesia in hands and feet	<148	N/A	N/A	N/A	Initially, 1 mg IM hydroxocobalamin three times a week for two weeks. On discharge, 1 mg IM hydroxocobalamin once every three months.	2	1

## Discussion

All the patients in this case series presented with neurological symptoms and signs secondary to N_2_O misuse with biochemical evidence of functional B12 deficiency. Despite this, in half the cases, the measured B12 concentration was within the normal reference range. Where measured, either the homocysteine, MMA levels or both were markedly raised even in those with a normal vitamin B12 level, highlighting the importance of a full and proper evaluation of a patient's vitamin B12 status using these additional markers in cases with documented neurological deficit in the presence of N2O misuse [5]. All patients received different B12 replacement regimens, although half the patients received alternate-day IM hydroxocobalamin for the first two weeks, highlighting the lack of standardised treatment guidelines in these patients. The absence of evidence-based guidance for reference and the fact that physicians, often with variable experience and knowledge of the pathology and treatment of N_2_O misuse, are left to rely on their clinical judgement when treating these patients highlight how vast variations in treatment regimens may occur.

The first case of functional B12 deficiency secondary to N_2_O misuse was documented over 40 years ago, and although numerous treatment schedules have been proposed, clear treatment guidelines are still lacking [6,7]. Abstinence from N_2_O and replacement of vitamin B12 are universally recognised as cores of treatment [8]. However, our case series has highlighted the lack of standardisation in treatment regarding dose, frequency or duration of vitamin B12 replacement. In addition, although treatment adjuncts such as folic acid and methionine have been advocated, no clear evidence or consensus around their use exists. Our case series highlights these treatment inconsistencies. Despite searching all neurological admissions over five years, we could only find six cases related to N_2_O toxicity. Since this time, there has been a marked increase in admissions related to N_2_O, and several cases were likely missed in our search due to a lack of consistency with the coding of electronic health records. When considering the limited sample size of this case series, further expansion of this trial and analysis from other centres in the United Kingdom and around the world would help quantify the prevalence of treatment inconsistencies in patients with N_2_O neurotoxicity and inform a standardised treatment regimen. 

Advice available to clinicians regarding the treatment of neurological sequelae secondary to N_2_O misuse remains inadequate, with current guidance particularly lacking in detail regarding the frequency and duration of treatment. The online database of the National Poisons Information Service (TOXBASE®) currently advises patients presenting with neurological features secondary to N_2_O use should receive 1 mg IM hydroxocobalamin alongside folic acid 5 mg orally, yet no information regarding recommended duration or frequency is given at the time of writing [9]. The National Institute of Health and Care Excellence (NICE) offers guidance on the treatment of vitamin B12 deficiency with neurological involvement but it is not directly translatable to the treatment of functional B12 deficiency seen in N_2_O misuse, given the different pathophysiology and symptomatology [10].

Published studies to date lack detail in terms of treatment regimen, the chronology of recovery within the studied period and whether patients remained abstinent. Studies have shown huge variability in recovery outcomes. A 2006 review of 45 observational studies involving patients diagnosed with SCDC secondary to N_2_O misuse showed that only 14% of patients achieved full symptomatic resolution with B12 therapy treatment over 20 months, with varying degrees of partial recovery seen in the remaining cohort [11-16]. 

Further complicating the assessment of treatment regimens is the lack of a gold-standard biochemical test of B12 status. Given that N_2_O misuse causes a functional rather than an absolute B12 deficiency, standard B12 assays add little in assessing disease resolution [17,18]. Homocysteine and MMA, although more accurate indicators of cellular B12 deficiency, offer no prognostic value, with a poor correlation between normalised values and functional neurological improvement following B12 replacement [19]. Randomised controlled trials (RCTs) with standardised treatment regimens and measurements of functional B12 status would be beneficial in creating a robust set of guidelines for clinicians to follow. However, such trials will be costly and time-consuming, and there is a more pressing need to create consensus guidelines. The authors propose initial treatment with IM hydroxocobalamin 1 mg on alternate days for two weeks, followed by reassessment. If symptoms improve, alternate-day treatment can be continued until improvement plateaus. If symptoms do not improve, further scrutiny into abstinence and differential diagnosis is required.

## Conclusions

Chronic N_2_O misuse results in functional B12 deficiency, most commonly manifesting as neurological complications, ranging from myelopathy to subacute combined degeneration of the spinal cord. Immediate abstinence from N_2_O is strongly advised with IM replacement of vitamin B12. Our case series highlights the current lack of consensus around treatment for such patients. As case numbers increase, having clear guidelines becomes increasingly important to improve care. There is a distinct need for RCTs to be conducted to help establish evidence-based guidelines for the treatment of N_2_O-induced myeloneuropathy through the assessment of optimal doses, forms and routes of vitamin B12 replacement, as well as other adjunctive treatments.
